# Research on the Fatigue Crack Growth Behavior of a Zr/Ti/Steel Composite Plate with a Crack Normal to the Interface

**DOI:** 10.3390/ma16155282

**Published:** 2023-07-27

**Authors:** Binbin Zhou, Jie Yuan, Haichao Song, Liangfu Zhou, Le Chang, Changyu Zhou, Cheng Ye, Bojun Zhang

**Affiliations:** 1 Engineering Technology Training Center, Nanjing Vocational University of Industry Technology, Nanjing 210023, China; 2 State Key Laboratory of Organic Electronics and Information Displays & Institute of Advanced Materials (IAM), Nanjing 210023, China; 3 School of Mechanical and Power Engineering, Nanjing Tech University, Nanjing 211816, China; 4 Technology Research and Development Department, Nanjing Boiler and Pressure Vessel Inspection Institute, Nanjing 210028, China

**Keywords:** fatigue crack growth, composite plate, loading parameters, equivalent plastic strain, elastic–plastic mismatch

## Abstract

The current work reveals the influence of loading parameters on the crack growth behavior of a Zr/Ti/steel composite plate with a crack normal to the interface by using an experiment and the finite element method. The Chaboche model was first used to study cyclic plastic evolution in composite materials. The results reveal that an increase in *F*_max_, *F*_m,_ and *F*_a_ can promote d*a*/d*N*; meanwhile, an increase in *R* will reduce d*a*/d*N.* The plastic strain accumulation results indicate that *F*_m_ mainly contributes to the tensile strain and compressive stress after the first cycle. Additionally, *F*_a_ increases the stress range and compression stress and greatly improves the plastic strain accumulation degree in subsequent loading cycles. The *F*_max_ can significantly increase the stress amplitude and plastic strain accumulation level. When *R* increases, the plastic strain accumulation increases a little, but the stress amplitude and compression stress decrease greatly. Furthermore, it is also found that the elastic–plastic mismatch also affects the plastic evolution, that is, strengthening or weakening the effect of the loading parameters.

## 1. Introduction

A Zr/Ti/steel composite plate uses zirconium as the cladding material, steel as the base material, and titanium as the transition layer [1,2]. A Zr/Ti/steel composite plate has excellent corrosion resistance, high strength, and certain plasticity; that is to say, it has good comprehensive performance and reduces the cost of manufacturing pure zirconium/titanium equipment. Therefore, it is widely used in the salt-making, metallurgy, chemical, and pharmaceutical industries [3].

There are generally differences in the elastic, plastic, and thermodynamic properties of materials composed of composite plates, which complicate the crack growth behavior in composites [4]. At present, the propagation behavior of cracks normal to the interface in composite material plates is receiving increasing attention. It is generally believed that when cracks propagate from one side of the material to the other, property mismatch will significantly affect their propagation behavior [5,6,7]. For an elastic mismatch, many scholars have put forward a variety of expressions for the *K* of this type of crack. Cirello [8] proposed the actual dimensionless K for sandwich materials. Zhong [9] used the interface layer model to establish a more accurate mechanical model by giving specific geometric dimensions to non-uniform thin layers and assuming that the material properties inside the layers are continuously changing. However, these expressions are limited to specific structural forms and various assumptions, resulting in the application scenarios being limited. Therefore, the finite element method has become the most widely used method to obtain *K*. For plastic mismatches, the inhomogeneous term method is used by Sistaninia [10], which can clarify the influence of elastic and plastic mismatches on far-field and near-field driving forces at the crack tip. Unfortunately, it is difficult to establish a relationship with d*a*/d*N*; therefore, its application is limited.

The influence of the loading parameters on d*a*/d*N* has been widely researched [11]. Based on the linear elastic fracture theory, the crack is modulated by *K*. Therefore, the effect of the loading parameters can be studied by their influence on *K* [12]. The peak load *F*_max_ increases the tensile strain and the static damage degree. The bending fatigue test results of Corigliano [13] showed that, with an increase in *F*_max_, cracks start from the aluminum alloy side and propagate towards the pure aluminum intermediate layer along the thickness direction. After reaching the interface, some cracks propagate in a different direction, resulting in delamination at the interface; meanwhile, another part continues to propagate towards the steel side through the interface. The stress amplitude directly increases *K*, and the load ratio *R* is mainly related to the crack closure effect [14]. Chandran [15] found that although mean load *F*_m_ does not change *K*, it changes *R* and has a resulting closure effect. Zhang [16] studied the fatigue cracks in forged Ti-Al-4V composite plates, with a focus on the effects of residual stress. It was found that the residual stress contributes to *F*_max_, which determines the initial crack initiation position and the propagation direction. Mayer [17] studied the damage mechanism, i.e., microstructure characteristics after failure, and analyzed the influence of *F*_m_. It is one-sided to explain the influence of the parameters on the crack growth behavior based on linear elastic fracture theory. In fact, with the help of the finite element method, considering cyclic loading, Li [18] studied the plastic deformation characteristics, damage accumulation, and energy-change process of materials under cyclic loading and analyzed the influence of four kinds of working conditions on crack growth.

So far, the influence of loading parameters on the plastic deformation behavior of single materials has been clarified; this effect is clearly different in composite materials, which has aroused our interest. In this paper, by using the finite element method and considering static and cyclic loading, the influence of loading parameters on the monotonic plastic deformation and cyclic plastic deformation damage of a Zr/Ti/steel composite plate is analyzed. In addition, the Chaboche model, considering cyclic plastic behavior, is first used to study composite materials, and the difference between crack tip deformation in single homogeneous materials and composite materials is discussed, clarifying the influence of property mismatch on cyclic characteristics.

## 2. Materials and Methods

### 2.1. Materials

A Zr/Ti/steel explosive welding composite plate was used in this study, in which zirconium and titanium were commercially pure, whereas the brand of steel was Q345R. Table 1 lists the basic mechanical properties of the three component materials, which were obtained via the tensile testing of the component materials. The elastic modulus of titanium and zirconium were very close, whereas their tensile strengths were very different. The thicknesses of each of the layers of the Zr/Ti/steel composite plate were 2, 1.8, and 8.2 mm, respectively. The composite plate was subjected to post-weld heat treatment; thus, residual stress was not considered in this study.

### 2.2. Fatigue Crack Growth Test

**Figure 1:**
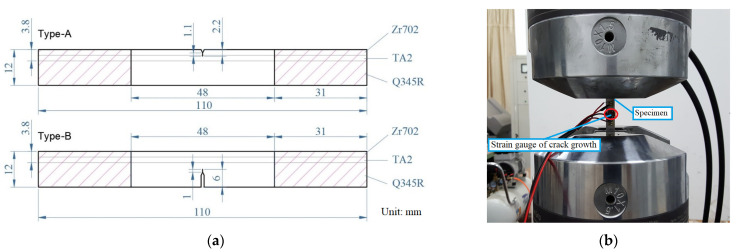
(**a**) Schematic diagram of Type-A and Type-B specimens; (**b**) fatigue crack growth test.

Considering the difference in the crack initiation location, two specimen types were considered, as shown in Figure 1a; the thicknesses of the Zr702, TA2, and Q345R layers were 2, 1.8, and 8.2 mm, respectively. The crack of the Type-A specimen propagated from ‘soft’ material to ‘hard’ material, whereas the crack of the Type-B specimen propagated from ‘hard’ material to ‘soft’ material. According to ASTM E647 [19], which requires the size of a SENT (single edge notch specimen), the specimen thickness is half of its width, i.e., 6 mm. Prior to the formal crack growth test, an initial crack prefabrication test was carried out on the specimens under fatigue loading. For the Type-A specimen, the crack length after prefabrication was 2.2 mm, whereas that of the Type-B specimen was 6 mm, as shown in Figure 1a. The initial crack prefabrication test was conducted according to ASTM E647, and its load was much smaller than that of the formal crack growth test. The test was carried out on MTS809 hydraulic testing machine at ambient temperature, as shown in Figure 1b. The shaded part of the sample (as shown in Figure 1) was clamped and subjected to stress-controlled fatigue testing. A formal crack growth test was carried out at 8 Hz, and a triangular wave was used. The specific loading parameters are shown in Table 2. As shown in Table 2, there were 9 groups for Type-A and 9 groups for Type-B.

**Figure 2:**
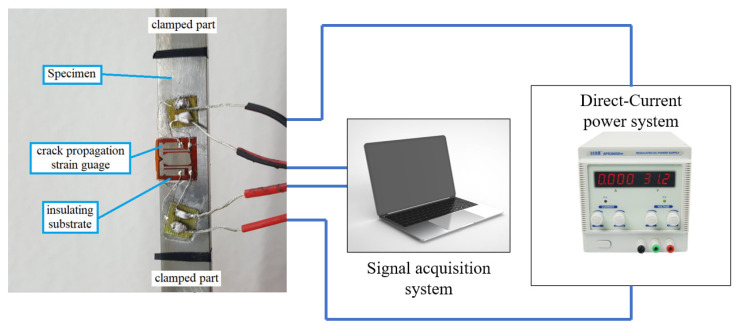
Schematic diagram of the direct current potential method.

Figure 2 shows that the direct current potential method [20] was used to measure the crack length. At the same time, the corresponding number of loading cycles was recorded. During the test, the load was automatically controlled and recorded by the MTS809 machine. After the test, the seven points polynomial method [21] was used to obtain the d*a*/d*N* data.

### 2.3. Finite Element Method

#### 2.3.1. Static Finite Element Method

**Figure 3:**
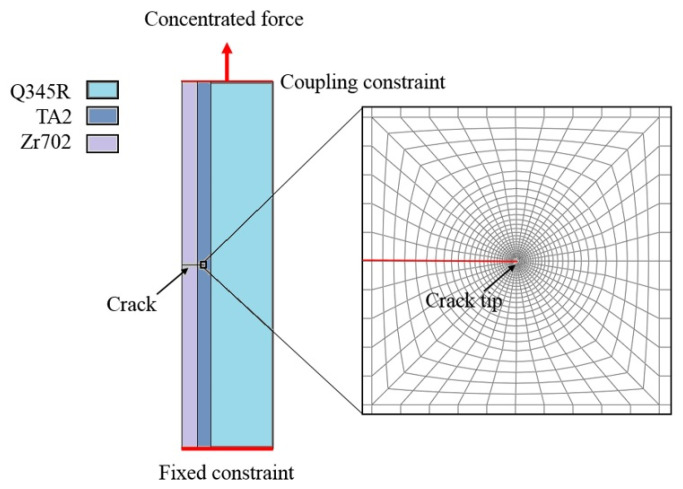
Finite element model of Type-A specimen.

The finite element software, ABAQUS, was used to obtain the monotonic plastic zone size of the crack tip. A two-dimensional finite element model was adopted here, and the element type was a plane strain element (CPE4). The material properties are defined in Table 1, and the boundary conditions and finite elements are shown in Figure 3 (Type-A crack as an example). 

As shown in Figure 3, one side of the specimen was set as a fixed constraint, the other side was a binding constraint, and a concentrated load was applied. At the same time, the other degrees of freedom of this side were limited, except in the direction of the concentrated force. The mesh of the crack tip was refined using linearized mesh generation in order to measure the size of the plastic zone conveniently. The minimum size was 0.001 mm and the maximum size was 0.01 mm. In this paper, the plastic zone was defined as the region in which the plastic strain was more than 10^−4^.

#### 2.3.2. The Finite Element Method Considering Cyclic Loading by Using the Chaboche Model

In order to describe the plastic deformation behavior of materials under cyclic loading, follow-up strengthening was considered [21]. By using the Chaboche model, which combines the criteria of follow-up strengthening and isotropic strengthening, ABAQUS was used to study the cyclic response, plastic deformation, and strain accumulation under cyclic loading in this study. 

According to the von Mises yield criterion, the yield surface of the Chaboche model is expressed as follows [22]:

(1) $$f\left(\sigma -\alpha ,k\right)=\sqrt{\frac{2}{3}\left(s-a\right)\cdot \left(s-a\right)}-k=0$$

In the above equation, σ is the stress tensor; α is the back stress tensor, i.e., the center of yield surface; s is the partial stress tensor; a is the partial back stress tensor; k is the yield surface size.

To describe the plastic flow direction, the plastic strain increment is expressed as follows:

(2) $$d\varepsilon^{p}=\lambda \frac{\partial f}{\partial \sigma }$$

where λ is the plastic multiplier. The following hardening law of the model is expressed as follows:

(3) $$d\alpha =\sum_{i=1}^{M}d\alpha_{i}$$

(4) $$d\alpha_{i}=\frac{2}{3}C_{i}d\varepsilon^{p}-\gamma_{i}\alpha_{i}dp$$

Here, $C_{i}$ and $\gamma_{i}$ are material parameters; dp is the cumulative plastic strain. The material parameters of each component material were obtained via uniaxial tensile testing of each homogeneous material, and the strengthening parameters were obtained via a low-cycle fatigue test. The parameters of the component materials and the data of isotropic strengthening were applied to the Chaboche model, whereas boundary conditions and constraints similar to those of the static finite element method were adopted. The parameters of the Chaboche model for each component are shown in Table 3. The parameters of the Chaboche model and the equivalent plastic strength–strain curve of the corresponding component material were obtained via basic tensile tests and low-cycle fatigue tests of the specimen. 

The experimental data used for finite element analysis in this study consisted of two parts: the first part was quasi-static data, which included the elastic and plastic results in the tensile stress–strain curve used for the quasi-static deformation simulation of finite element analysis. The second part was a low-cycle fatigue test in order to obtain the Chaboche model parameters and equivalent plastic strength–strain curve for dynamic fatigue simulation.

## 3. Results and Discussion

### 3.1. The Influence of Loading Parameters on Fatigue Crack Growth Behavior

#### 3.1.1. The Influence of Loading Parameters on d*a*/d*N*

**Figure 4:**
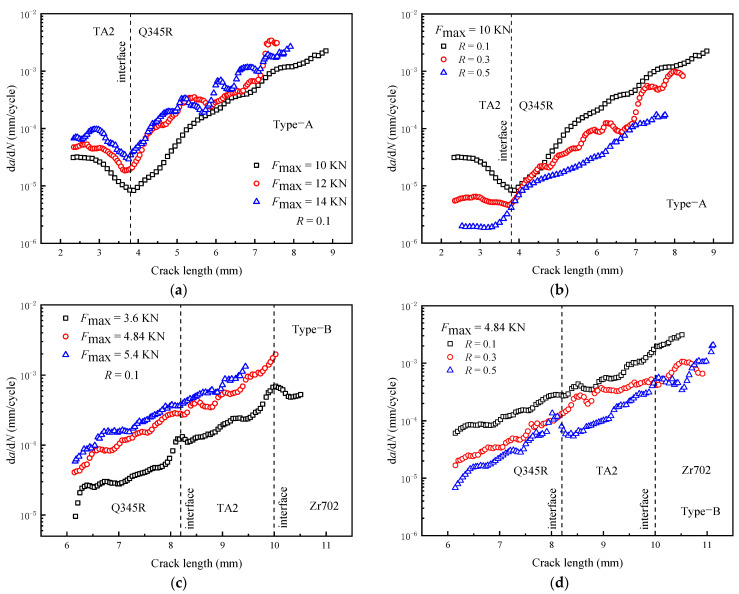
The effect of *F*_max_ and *R* on the d*a*/d*N* (experimental results): (**a**,**b**) Type-A; (**c**,**d**) Type-B.

Figure 4a,c show that the crack in the Type-A specimen is hindered by the property mismatch. An elastic mismatch results in a decrease in the driving force of the crack tip, further leading to a decrease in d*a*/d*N* near the interface. The d*a*/d*N* of the crack tip increases with *F*_max_, which is consistent with the variation pattern in homogeneous materials. Obviously, when *F*_max_ increases, Δ*K* increases, thus increasing d*a*/d*N*. Figure 4b,d show that d*a*/d*N* decreases with an increase in *R*. In Figure 4b, the d*a*/d*N* results under different *R* values are similar at the interface, but the gap gradually increases as the crack continues to expand at the Q345R side. The results show that the d*a*/d*N* of each side decreases with an increase in *R*, so it can be considered that Δ*K* is the main factor affecting d*a*/d*N*. Secondly, due to the significant differences in the properties and resistance to crack growth of component materials, the variation in Δ*K* affected by the elastic mismatch and the propagation characteristics of cracks in homogeneous single materials should be considered in d*a*/d*N* analysis near the interface.

**Figure 5:**
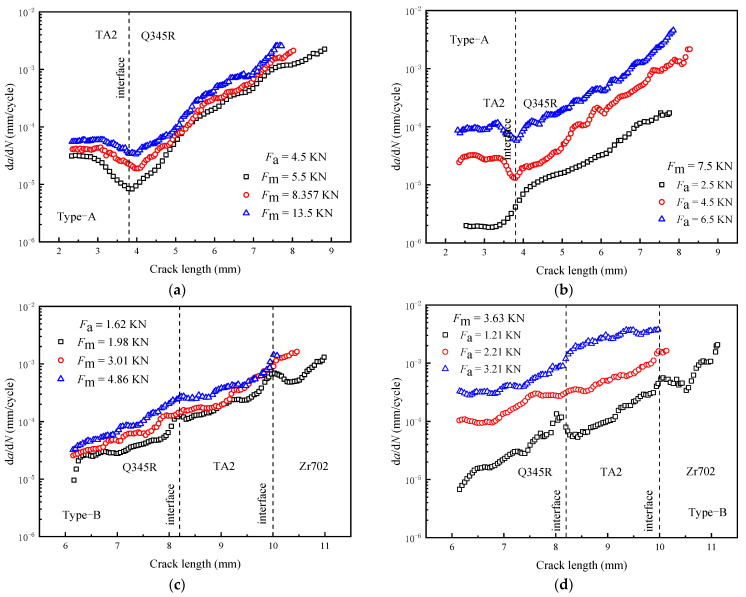
The effect of *F*_m_ and *F*_a_ on d*a*/d*N* (experimental results): (**a**,**b**) Type-A; (**c**,**d**) Type-B.

The influence of *F*_m_ and the load amplitude *F*_a_ on d*a*/d*N* is shown in Figure 5. Figure 5a,c show that the d*a*/d*N* of Type-A and Type-B cracks increases with *F*_m_, but this increase is limited. Actually, *F*_m_ does not affect the driving force of the crack, and its effect on d*a*/d*N* is mainly achieved by improving the damage degree of the crack tip. In the same way, due to the elastic–plastic mismatch of the Type-A specimen, the results of the d*a*/d*N* under different *F*_m_ values first decrease and then increase near the interface. In contrast, the change in d*a*/d*N* at the interface of the Type-B specimen can be ignored. Figure 5b,d show that an increase in *F*_a_ can greatly promote d*a*/d*N*. This is because *F*_a_ directly determines the driving force, Δ*K*, to improve d*a*/d*N*. 

#### 3.1.2. The Influence of Loading Parameters on the Plastic Zone of Crack Tip

There are two main types of damage in the process of fatigue crack growth: static damage and cyclic damage [18]. The static damage is controlled by the maximum stress intensity factor *K*_max_, whereas the cyclic damage is controlled by Δ*K*. In view of the linear elastic fracture mechanics, the monotonic plastic zone can be determined by *K*_max_, whereas Δ*K* under cyclic loading depends on *F*_a_. Therefore, for a monotonic plastic zone under cyclic loading, *F*_a_ is the most critical parameter. Because the quasi-static model cannot consider the plastic strain accumulation of the crack tip, this section mainly studies the influence of loading parameters on the monotonic damage degree by studying the change in the monotonic plastic zone.

**Figure 6:**
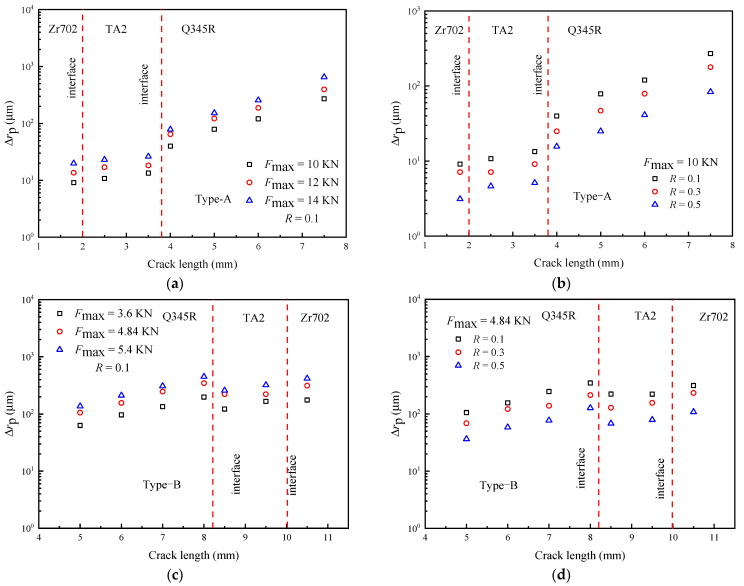
The effect of *F*_max_ and *R* on the change of plastic zone (simulation results): (**a**,**b**) Type-A; (**c**,**d**) Type-B.

Figure 6 shows the influence of *F*_max_ and *R* on the monotonic plastic zone change for Type-A and Type-B specimens. Figure 6a,c show that when *R* is fixed, plastic deformation increases with *F*_max_. At this time, *F*_max_ leads to the synchronous rise of *F*_m_ and *F*_a_, so the crack tip bears a higher stress level, thus improving the plastic deformation degree. As shown in Figure 6b,d, when *F*_max_ is fixed, the monotonic plastic deformation decreases significantly with an increase of *R*. At this time, because *F*_max_ is constant, *F*_a_ decreases with an increase in *R*. Relative to *F*_m_, *F*_a_ plays a dominant role in monotonic plastic deformation, and the changing trend of the monotonic plastic deformation level at the crack tip is consistent with that of *F*_a_.

**Figure 7:**
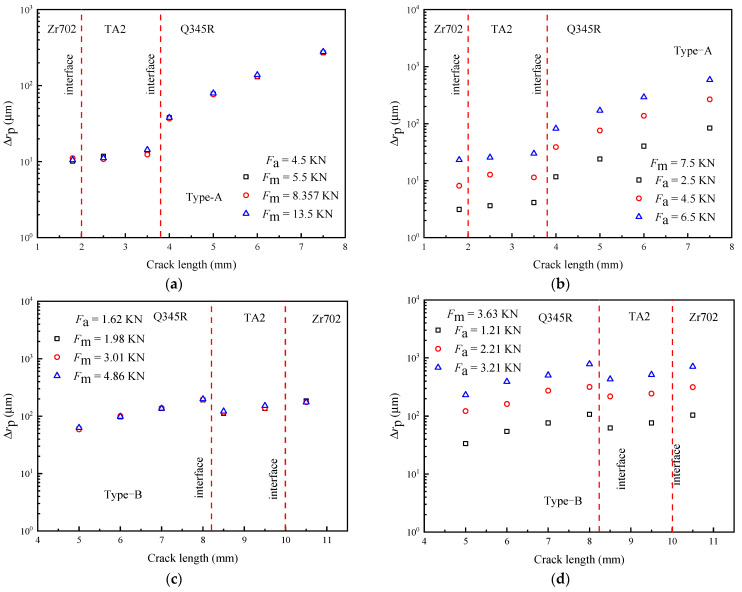
The effect of *F*_m_ and *F*_a_ on the change of plastic zone (simulation results): (**a**,**b**) Type-A; (**c**,**d**) Type-B.

Figure 7 shows the influence of *F*_m_ and *F*_a_ on monotonic plastic zone change. As shown in Figure 7a,c, the static finite element simulation results show that an increase in *F*_m_ does not change the monotonic plastic deformation. In the static finite element method, *F*_a_ is the same under different *F*_m_ values, so the monotonic plastic zone change is the same. It can be seen that using the static finite element to describe the influence of *F*_m_ on crack growth behavior has limitations because the influence of *F*_m_ needs to be analyzed by considering the cyclic deformation characteristics of the finite element considering cyclic loading. The monotonic plastic zone change results under different *F*_a_ values (Figure 7b,d) show that when *F*_m_ remains unchanged, *F*_a_ significantly improves the plastic deformation ability of the crack tip.

### 3.2. The Influence of Loading Parameters on Cyclic Plastic Deformation

The influence of the loading parameters on the crack growth behavior can be studied from the cyclic deformation [23]. In this section, the hysteresis curve is used to describe the cyclic deformation behavior and to explore the influence of the loading parameters on plastic deformation.

**Figure 8:**
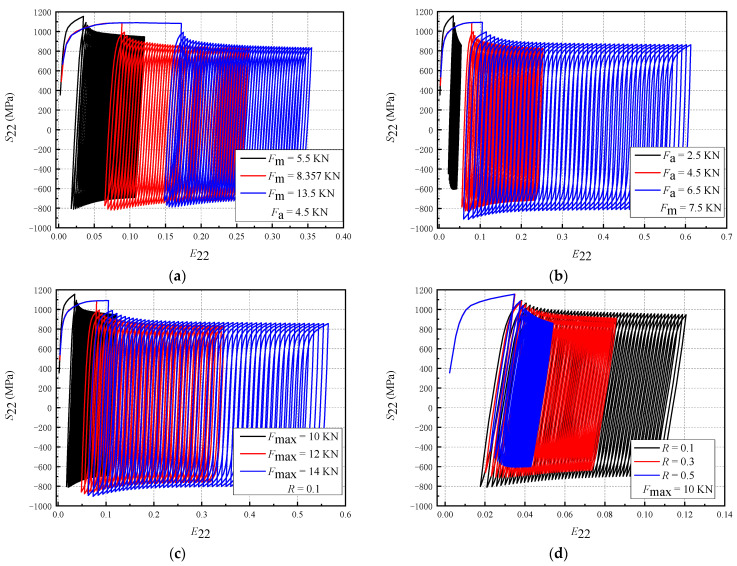
The effect of loading parameters on the cyclic plastic deformation (single homogeneous material, simulation results): (**a**) *F*_m_; (**b**) *F*_a_; (**c**) *F*_max_; (**d**) *R*.

For a composite structure, the cyclic deformation behavior is affected by the properties mismatch of the component materials. Therefore, a single homogeneous material was studied first. Figure 8 shows the influence of the loading parameters on the cyclic deformation behavior. The size of the homogeneous material specimen studied here is the same as that of the composite plate specimen. Material TA2 is taken, and the crack length is 3.5 mm; meanwhile, the response curves of stress S_22_ and strain E_22_ under 50 cycles are taken 5 μm away from the crack tip. 

The non-zero mean stress exists in the material under asymmetrical load, which leads to plastic strain accumulation and ratcheting deformation. Figure 8a shows that the tensile strain after the first cycle of loading increases significantly with *F*_m_, whereas the strain accumulation under the subsequent loading cycle is also affected by *F*_m_. Therefore, *F*_m_ mainly affects the tensile strain after the first loading cycle. In Figure 8b, when *F*_m_ is constant, the stress range and the compression stress increase with *F*_a_, and the accumulation degree of plastic strain increases significantly in the subsequent periods. Figure 8c shows that when *R* is constant and *F*_max_ is increased, *F*_m_ and *F*_a_ also increase, thereby increasing the stress amplitude and the accumulated tensile strain. Additionally, with an increase in *F*_max_, the compressive stress also increases. When *F*_max_ is the same and *R* is different, *F*_m_ increases and *F*_a_ decreases with an increase in *R*. It can be seen from Figure 8d that the increase in *R* leads to a significant decrease in the stress amplitude and compression stress and a significant decrease in the strain accumulation. Compared with the decrease in the cumulative plastic strain caused by *F*_a_, the effect of *F*_m_ on the hysteresis curve is not significant. Therefore, d*a*/d*N* is greatly reduced.

**Figure 9:**
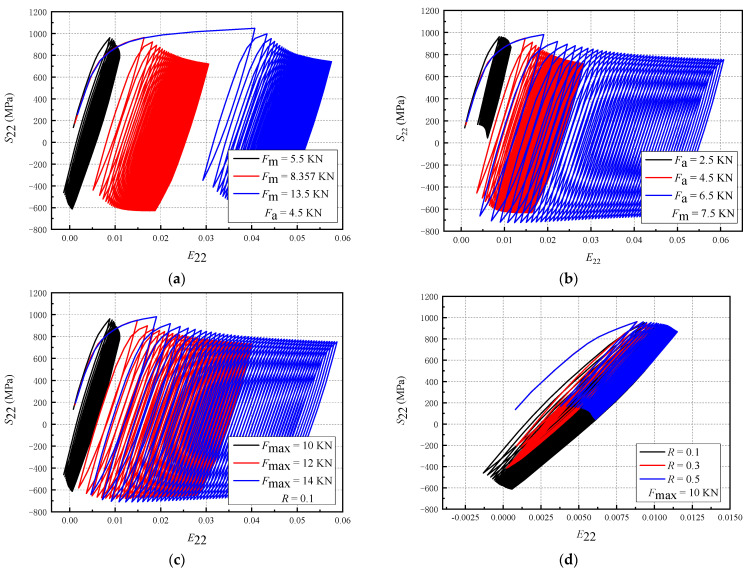
The effect of loading parameters on cyclic plastic deformation (Type-A crack, simulation results): (**a**) *F*_m_; (**b**) *F*_a_; (**c**) *F*_max_; (**d**) *R*.

Therefore, the influence of loading parameters on the cyclic stress–strain behavior of a single homogeneous material can be summarized as follows: (1) *F*_m_ will significantly increase the tensile strain and reduce the compressive stress after the first loading cycle, and increase the plastic stress accumulation during the subsequent loading cycles; (2) *F*_a_ will increase the stress amplitude and the compressive stress, and significantly increase the following cumulative degree of cyclic plastic strain; (3) *F*_max_ will increase the level of stress amplitude and the plastic strain accumulation, and its contribution to the crack growth has the characteristics of *F*_m_ and *F*_a_; (4) *R* will lead to a decrease in *F*_a_ and an increase in *F*_m_. Compared to the increase in *F*_m_, the decrease in cumulative plastic strain caused by the decrease in *F*_a_ has a stronger effect on crack growth. In order to consider the effect of property mismatch, this paper also studied the cyclic deformation behavior of composite materials. Here, the Type-A specimen was considered, and the crack length was 3.5 mm. The response curves of stress S_22_ and strain E_22_ in 50 cycles at 5 μm from the crack tip are shown in Figure 9.

By comparing the cyclic deformation results of a single homogeneous material and the composite material, the following conclusions can be drawn. (1) Due to the elastic mismatch, the actual stress level of the Type-A specimen is lower than that of a single homogeneous material under the corresponding load when the crack does not pass the TA2/Q345R interface, so the stress amplitude decreases. (2) Under the same load level, the strain accumulation and the plastic deformation level are greatly reduced. When a crack propagates from the ‘soft’ component to the ‘hard’ component, a property mismatch will lead to a significant reduction in the plastic strain accumulation and damage level, thus further reducing d*a*/d*N*. (3) The influence of the loading parameters on the cyclic deformation behavior of the composite material is similar to that of a single homogeneous material, but the degree of influence is affected by the property mismatch.

Based on the above conclusion, the following reasonable assumptions were made: by adding a composite coating to a single substrate, the crack propagation problem in a single material becomes the crack propagation problem in the composite material. In addition, by cleverly utilizing the differences in material properties, the driving force for the crack propagation can be effectively reduced, and the accumulation of plastic damage at the crack tip can be reduced to achieve the purpose of protecting the base material.

### 3.3. The Effect of Loading Parameters on Equivalent Plastic Strain

**Figure 10:**
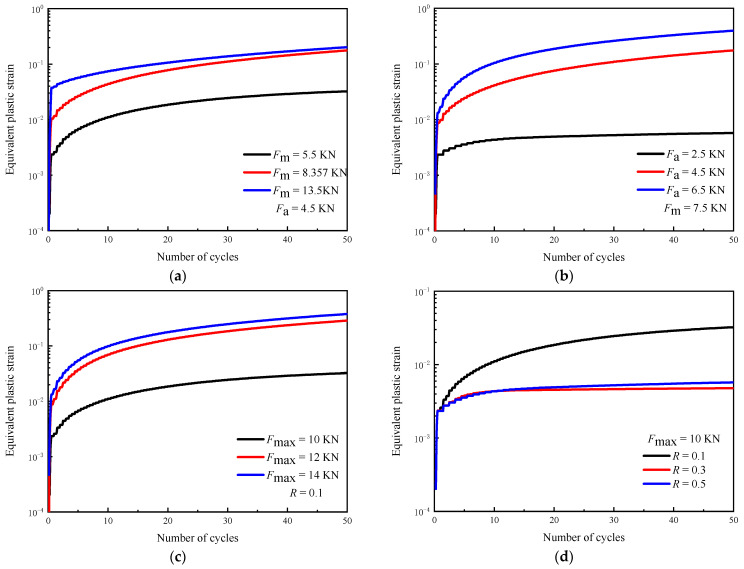
The effect of loading parameters on equivalent plastic strain (simulation results): (**a**) *F*_m_; (**b**) *F*_a_; (**c**) *F*_max_; (**d**) *R*.

Figure 10 shows the equivalent plastic strain of the first 50 cycles at 5 μm from the crack tip. It can be seen that the equivalent plastic strain keeps accumulating with the cycle number, and the accumulation speed shows a trend of changing from fast to slow under different loading parameters, but there are significant differences. Considering the changing trend of the hysteresis loop under different loading parameters, the influence of *F*_m_ and *F*_a_ on the equivalent plastic strain is analyzed. Figure 10a shows that the equivalent plastic strain increases rapidly in the first loading cycle with *F*_m_, especially when *F*_m_ is 13.5 kN, which is consistent with the change rule of E_22_ (Figure 8a). The last 20 loading cycles show that the effect of *F*_m_ on the equivalent plastic strain under subsequent loading cycles is limited, and the accumulation rate of the equivalent plastic strain under different *F*_m_ values is close. Figure 10b shows that an increase in *F*_a_ causes the accumulation of plastic strain under each loading cycle, and the accumulation speed of the plastic strain increases with the loading cycle. This is different from *F*_m_, which mainly affects the tensile plastic strain under the first cycle.

In Figure 10c, *F*_max_ has the same effect on the equivalent plastic strain during the whole loading process, and its effect on the plastic strain accumulation in the subsequent loading cycle is significantly lower than that of *F*_a_. The increase in *F*_max_ increases *F*_m_ and *F*_a_, so its influence on the plastic strain should have the characteristics of *F*_m_ and *F*_a_. Accordingly, it can be seen from Figure 10d that an increase in *R* greatly reduces plastic strain accumulation. Although *F*_m_ increases, the decrease in *F*_a_ results in a decrease in the plastic strain accumulation at each loading cycle. In addition, the results of *R* = 0.3 and *R* = 0.5 are similar, which shows that the influence of *F*_a_ and *F*_m_ on plastic deformation is interactive.

**Figure 11:**
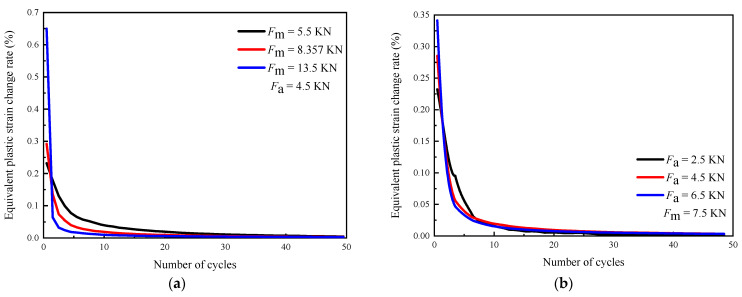

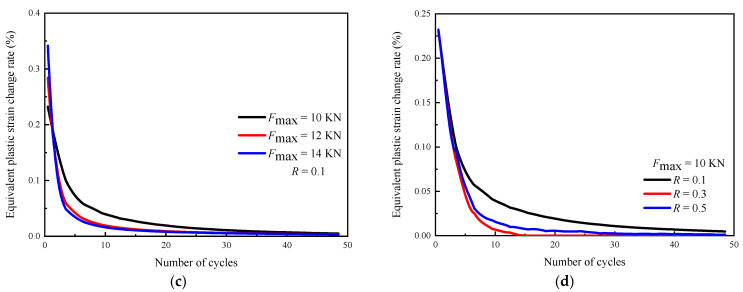
The effect of loading parameters on equivalent plastic strain change rate (simulation results): (**a**) *F*_m_; (**b**) *F*_a_; (**c**) *F*_max_; (**d**) *R*.

By comparing the change rate of the equivalent plastic strain, the influence of the loading parameters on the cumulative rate of plastic deformation is more obvious, as shown in Figure 11. It can be seen from Figure 11a that *F*_m_ greatly improves the plastic strain accumulation rate under the first loading cycle. As the loading proceeds, the plastic strain accumulation rate decreases with *F*_m_, and the influence of *F*_m_ is smaller. Figure 11b shows that the plastic strain accumulation of the first loading cycle is slightly increased by *F*_a_. With the increase in loading cycles, the plastic strain accumulation rate decreases with *F*_a_, and the plastic strain accumulation rate under different *F*_a_ values is similar. As shown in Figure 11c, the plastic strain accumulation rate under the first loading cycle increases with *F*_max_ and then decreases with the loading cycles. In addition, the higher the *F*_max_, the lower the plastic strain accumulation rate under the subsequent loading cycle. When *F*_m_, *F*_a,_ and *F*_max_ increase, the plastic deformation change after the first loading is the largest, and the subsequent plastic deformation ability of the material decreases, so the plastic strain accumulation rate is reduced under the subsequent loading cycle. Figure 11d shows the effect of *R* on the change rate of the equivalent plastic strain 5 μm from the crack tip. It can be seen from Figure 11a,b that the increase in *F*_m_ and *F*_a_ reduces the plastic strain change rate under the subsequent loading cycle. When *R* increases, *F*_m_ also increases while *F*_a_ decreases. Therefore, the influences of *F*_m_ and *F*_a_ on the plastic strain change rate under the subsequent loading cycle interact with each other. In this case, *F*_m_ and *F*_a_ change within the same range. The results show that when *R* increases, the influence of *F*_m_ and *F*_a_ on the plastic strain rate of change under the first loading cycle is mutually offset, so the plastic strain rate change is constant. In the subsequent loading cycle, the results of *R* = 0.5 and *R* = 0.3 are lower than *R* = 0.1, which shows that the decrease in the plastic strain rate due to the increase in *F*_m_ is greater than the increase in the plastic strain rate due to the decrease in *F*_a_.

The plastic strain accumulation in the first loading cycle is determined by *F*_max_. When *F*_max_ is fixed, the plastic strain accumulation in the first loading cycle is fixed. Then, in the subsequent cyclic loading, the increase in *F*_a_ and *F*_m_ increases the accumulation degree of the plastic strain. However, due to the premature consumption of the plastic deformation ability, the accumulation rate of the plastic strain decreases under the subsequent loading cycle.

**Figure 12:**
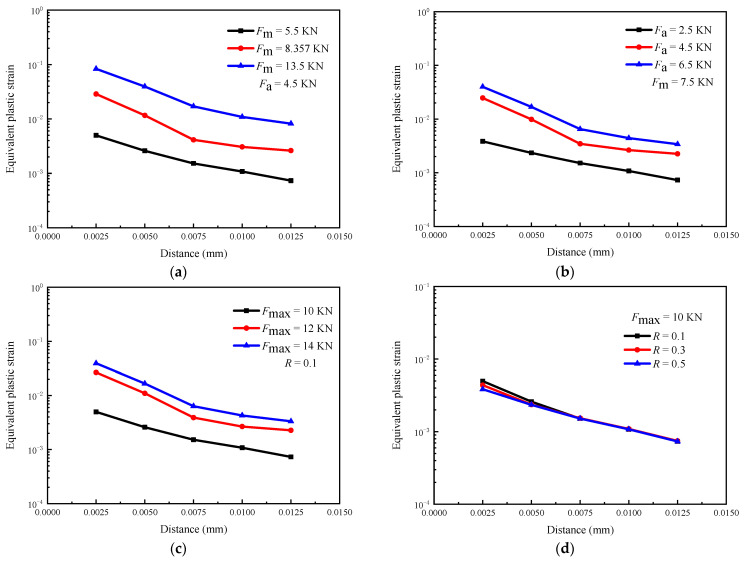
The effect of loading parameters on equivalent plastic strain distribution near the crack tip (simulation results): (**a**) *F*_m_; (**b**) *F*_a_; (**c**) *F*_max_; (**d**) *R*.

Figure 12 shows the effect of the loading parameters on the equivalent plastic strain distribution near the crack tip after the first loading cycle. Figure 12a–c show that the plastic deformation level increases with *F*_m_, *F*_a,_ and *F*_max_, and decreases with the distance from the crack tip. In addition, the influence of these loading parameters on plastic deformation is not significantly related to the distance from the crack tip. Figure 12d shows that the plastic deformation level of the crack tip decreases with *R*, and the effect decreases with the increasing distance from the crack tip.

## 4. Conclusions

In this study, the Chaboche model is first used to study cyclic plastic evolution during crack propagation in composite materials. Based on the experimental results, the influence of the loading parameters on the crack propagation behavior of a Zr/Ti/steel composite plate is studied in detail, and the effect of property mismatch is investigated. The specific conclusions are as follows:

(1) An increase in *F*_max_, *F*_m,_ and *F*_a_ can promote d*a*/d*N*, whereas an increase in *R* can reduce d*a*/d*N*.

(2) An increase in *F*_m_ is mainly due to the tensile strain and compressive stress after the first cycle. *F*_a_ increases the stress range and compression stress and greatly improves the plastic strain accumulation degree of the subsequent loading cycles. Additionally, the *F*_max_ can significantly increase the stress amplitude and the plastic strain accumulation level. When *R* increases, the plastic strain accumulation increases a little, but the stress amplitude and the compression stress decrease greatly.

(3) The influence of the loading parameters on the cyclic deformation behavior of the composite material is similar to that of a single homogeneous material. In addition, the elastic–plastic mismatch also affects the plastic evolution, that is, strengthening or weakening the effect of the loading parameters.

It can be confirmed that the influence of loading parameters on the crack tip damage is influenced by the degree of difference between material properties, while this paper only studied the Type-A cracks (cracks from ‘soft’ materials to ‘hard’ materials), with a single property difference. Further research on the combination of different material property differences urgently needs to be carried out. We firmly believe that by cleverly utilizing the differences in material properties, the driving force for the crack propagation can be effectively reduced, and the accumulation of plastic damage at the crack tip can be reduced to achieve the purpose of protecting the base material.

## Figures and Tables

**Table 1:** Comparison of mechanical properties of Zr702, TA2, and Q345R.

Material	Elastic Modulus [GPa]	Poisson Ratio	Yield Strength [MPa]	Tensile Strength [MPa]
Zr702	90.3	0.33	314	499
TA2	90	0.34	330	383
Q345R	203	0.3	352	522

**Table 2:** Fatigue crack growth test.

Number	*F*_max_ [kN]	*R*	*F*_m_ [kN]	*F*_a_ [kN]
A-1	10	0.1	5.5	4.5
A-2	12	0.1	6.6	5.4
A-3	14	0.1	7.7	6.3
A-4	10	0.3	6.5	3.5
A-5	10	0.5	7.5	2.5
A-6	12.857	0.3	8.357	4.5
A-7	18	0.5	13.5	4.5
A-8	12	0.25	7.5	4.5
A-9	14	1/14	7.5	6.5
B-1	3.6	0.1	1.98	1.62
B-2	4.84	0.1	2.662	2.178
B-3	5.4	0.1	2.97	2.43
B-4	4.84	0.3	3.146	1.694
B-5	4.84	0.5	3.63	1.21
B-6	4.63	0.3	3.01	1.62
B-7	6.48	0.5	4.86	1.62
B-8	5.84	0.2432	3.63	2.21
B-9	6.84	0.0614	3.63	3.21

**Table 3:** Chaboche model parameters of the component materials.

Material	*σ*_0_	*C*_1_	*γ*_1_	*C*_2_	*γ*_2_
Zr702	314	1759	199	1758	18
TA2	330	2067	26	1765	24
Q345R	352	1752	20	2071	7

## Data Availability

Not applicable.
